# Cardiovascular adverse events associated with immune checkpoint inhibitors: A retrospective multicenter cohort study

**DOI:** 10.1002/cam4.7233

**Published:** 2024-05-16

**Authors:** Yi Zheng, Ziliang Chen, Wenhua Song, Yu Xu, Zhiqiang Zhao, Yihong Sun, Yuanyuan Wang, Xuhong Geng, Jun Zhao, Xiaowei Zhang, Yanmin Xu, Jeffrey Shi Kai Chan, Gary Tse, Guangping Li, Lili Hong, Tong Liu

**Affiliations:** ^1^ Tianjin Key Laboratory of Ionic‐Molecular Function of Cardiovascular Disease, Department of Cardiology Tianjin Institute of Cardiology, Second Hospital of Tianjin Medical University Tianjin China; ^2^ Department of Oncology Tianjin Huanghe Hospital Tianjin China; ^3^ Department of Cardiology China‐Japan Friendship Hospital Beijing China; ^4^ Department of Function Fourth Hospital of Hebei Medical University Shijiazhuang Hebei China; ^5^ Cardio‐Oncology Research Unit Cardiovascular Analytics Group Hong Kong China; ^6^ School of Nursing and Health Studies Hong Kong Metropolitan University Hong Kong China; ^7^ Cardiac Electrophysiology Unit, Cardiovascular Analytics Group PowerHealth Limited Hong Kong China

**Keywords:** cardiac troponin‐I, cardiovascular adverse events, creatine kinase isoenzyme‐MB, immune checkpoint inhibitor, myocarditis, neutrophil to lymphocyte ratio

## Abstract

**Background:**

Over the past decade, immune checkpoint inhibitors (ICIs) have significantly transformed cancer treatment. However, ICIs inevitably may cause a spectrum of immune‐related adverse events, among which cardiovascular toxicity, particularly myocarditis, while infrequent, has garnered increasing attention due to its high fatality rate.

**Methods:**

We conducted a multicenter retrospective study to characterize ICI‐associated cardiovascular adverse events. Logistic regression was performed to explore the risk factors for the development of myocarditis and severe myocarditis. Receiver operating characteristic curves were conducted to assess the diagnostic abilities of cardiac biomarkers to distinguish different cardiovascular toxicities, and the performance and calibration were evaluated using Hosmer–Lemeshow test.

**Results:**

Forty‐four patients were identified, including thirty‐five myocarditis, five heart failure, three arrhythmias, and one myocardial infarction. Compared with other patients, myocarditis patients had higher cardiac troponin‐I (cTnI) levels (*p* < 0.001), higher creatine kinase levels (*p* = 0.003), higher creatine kinase isoenzyme‐MB (CK‐MB) levels (*p* = 0.013), and shorter time to the incidence of adverse cardiovascular events (*p* = 0.022) after ICI treatment. Twenty‐one patients (60%) were classified as severe myocarditis, and they presented higher cardiac troponin I (cTnI) levels (*p* = 0.013), higher N‐terminal pro‐B‐type natriuretic peptide levels (*p* = 0.031), higher creatine kinase levels (*p* = 0.018), higher CK‐MB levels (*p* = 0.026), and higher neutrophil to lymphocyte ratio (NLR) levels (*p* = 0.016) compared to non‐severe myocarditis patients after ICI treatment. Multivariate logistic regression showed that CK‐MB (adjusted odds ratio [OR]: 1.775, 95% confidence interval [CI]: 1.055–2.984, *p* = 0.031) was the independent risk factor of the development of ICI‐associated myocarditis, and cTnI (adjusted OR: 1.021, 95% CI: 1.002–1.039, *p* = 0.03) and NLR (adjusted OR: 1.890, 95% CI: 1.026–3.483, *p* = 0.041) were the independent risk factors of ICI‐associated severe myocarditis. The receiver operating characteristic curve showed an area under curve of 0.785 (95% CI: 0.642 to 0.928, *p* = 0.013) for CK‐MB, 0.765 (95% CI: 0.601 to 0.929, *p* = 0.013) for cTnI, and 0.773 for NLR (95% CI: 0.597 to 0.948, *p* = 0.016).

**Conclusions:**

Elevated CK‐MB after ICI treatment is the independent risk factor for the incidence of ICI‐associated myocarditis, and elevated cTnI and NLR after ICI treatment are the independent risk factors for the development of ICI‐associated severe myocarditis. CK‐MB, cTnI, and NLR demonstrated a promising predictive utility for the identification of ICI‐associated myocarditis and severe myocarditis.

## 1 INTRODUCTION

Cardiovascular diseases and malignant tumors share many common risk factors and biological mechanisms, which promotes the emergence of cardio‐oncology. A recent study has suggested a positive association between cardiovascular diseases and the incidence of bladder cancer.^1^ Over the preceding decades, the landscape of cancer therapy has undergone a profound transformation, marked by the incorporation of antitumor immunity as a novel instrument in the therapeutic area. Immune checkpoints encompass a repertoire of cell‐surface‐expressed receptors that function as key components in the orchestration of negative immune regulatory processes. These receptors assume a pivotal responsibility in the preservation of immune homeostasis and the mitigation of autoimmunity.^2^ Immune checkpoint inhibitors (ICIs) are monoclonal antibodies against immune checkpoints, currently approved ICIs target four inhibitory immune checkpoints, including programmed cell death 1 (PD1), programmed cell death ligand 1, cytotoxic T lymphocyte‐associated antigen 4 (CTLA‐4), and lymphocyte activation gene‐3.^3^ Currently, ICIs have found application in the treatment of up to 50% of distinct cancer types.^4^ Nonetheless, the activation of the immune system through ICIs can precipitate immune‐mediated damage. Recent years have witnessed a proliferation of reports of immune‐related adverse events in cancer patients undergoing ICIs therapy, some of which proved fatal.^5^, ^6^, ^7^ Among these cases, while the incidence of adverse cardiovascular events induced by ICIs therapy remains relatively low, severe outcomes, including fatalities, have occurred in certain patients, especially myocarditis.^8^, ^9^

However, it has been only a few years for ICIs to be used clinically, and there is limited understanding of their cardiovascular toxicity. The comprehensive investigation and in‐depth analysis of the clinical characteristics, and underlying risk factors of ICI‐related myocarditis, as well as other adverse cardiac events, continue to be an area of active exploration and research within the medical and cardiology communities. Moreover, the diagnosis of ICI‐associated myocarditis is challenging, needing a combination of cardiac biomarkers (including cardiac troponin‐I [cTnI], creatine kinase [CK], and creatine kinase isoenzyme‐MB [CK‐MB]), imaging, and endomyocardial biopsy.^10^, ^11^ As we know, there are limited clinical data on the diagnosis of ICI‐associated myocarditis. The diagnostic performance of different cardiac biomarkers for the diagnosis and prediction of ICIs‐associated cardiovascular adverse events is still unknown.

This study is a retrospective multicenter cohort study, and the purpose of the study is to compare clinical data between ICI‐associated myocarditis versus non‐myocarditis immune‐related adverse events, and between ICI‐associated severe myocarditis versus non‐severe myocarditis including electrocardiography (ECG), echocardiography, cardiac biomarkers, and so forth. We further screened for risk factors for the development of myocarditis and severe myocarditis through statistical analysis, and explored their diagnostic ability.

## 2 METHODS

This retrospective multicenter cohort study included patients with ICIs‐associated cardiovascular adverse events at multiple medical centers including the Second Hospital of Tianjin Medical University, Tianjin Huanghe Hospital, Beijing China‐Japan Friendship Hospital and Fourth Hospital of Hebei Medical University from January 28, 2019 to June 15, 2023. This study was performed in accordance with the principles of the Declaration of Helsinki and the study protocol was approved by the Ethics Committee of the Second Hospital of Tianjin Medical University (KY2018K049). Among the reports of adverse cardiovascular events, cases were subjected to a filtering process, wherein only instances of cardiovascular adverse events associated with ICIs were retained for analysis (*n* = 44). This selection was contingent upon unanimous agreement among four independent adjudicators (Y.Z., Z.L.C., W.H.S, and T.L.), who assessed the cases based on pre‐established criteria and after excluding any potential underlying confounding factors. ICIs included were the following categories: (1) anti‐PD1 monotherapy; (2) combinations of anti‐PD1 with either chemotherapies or targeted therapies that included tyrosine kinase inhibitors or anti‐vascular endothelial growth factor (VEGF) agents; and (3) three treatment modalities that encompassed the concurrent administration of anti‐PD1 agents with chemoradiotherapy and targeted therapy. ICIs‐associated cardiovascular adverse events were categorized as: (1) myocarditis, (2) heart failure, (3) arrhythmias, and (4) myocardial infarction. Myocarditis definition was based on the International Cardio‐Oncology Society.^12^ All cases included in our study were definite myocarditis cases. Myocarditis severity was graded according to the American National Comprehensive Cancer Network.^13^ Concomitant non‐cardiac immune related adverse events were characterized by clinically, radiologically, or laboratory‐confirmed manifestations occurring within a temporal window of 4 weeks before or after the onset of ICI‐associated cardiovascular adverse events.^14^

An examination of medical records was conducted for demographic data, previous medical history, oncologic treatments, clinical data (time to cardiovascular adverse events occurrence, signs and symptoms), laboratory and imaging data after ICI treatment (cTnI, N‐terminal pro‐B‐type natriuretic peptide [NT‐proBNP], CK‐MB, D‐dimer, neutrophil to lymphocyte ratio [NLR], Albumin/globulin, aspartate transaminase (AST)/alanine transaminase [ALT], low‐density lipoprotein (LDL)/high‐density lipoprotein [HDL], ECG, and left ventricular ejection fraction [LVEF] measured by Simpson's biplane method). To ensure standardization, laboratory data values (cTnI, NT‐proBNP, CK, CK‐MB, and D‐dimer) were normalized according to the upper limit reference established by each respective laboratory.

Quantitative variables were presented in the form of medians along with their interquartile ranges (IQR) and compared with Mann–Whitney *U*‐tests. Qualitative variables were reported as percentages and assessed for differences using chi‐squared tests. Univariate and multivariate logistic regression were performed to analyze the risk factors for the development of ICI‐associated myocarditis in all included patients and severe ICI‐associated myocarditis in myocarditis patients. A receiver operating characteristic (ROC) curve was conducted to evaluate the diagnostic abilities of laboratory data to distinguish ICI‐associated myocarditis and severe myocarditis. Hosmer–Lemeshow tests were conducted to evaluate performance and calibration. Statistical differences were considered significant if *p* < 0.05. All statistical analysis was made using SPSS version 27.

## 3 RESULTS

### 3.1 Basic characteristics

**FIGURE 1:**
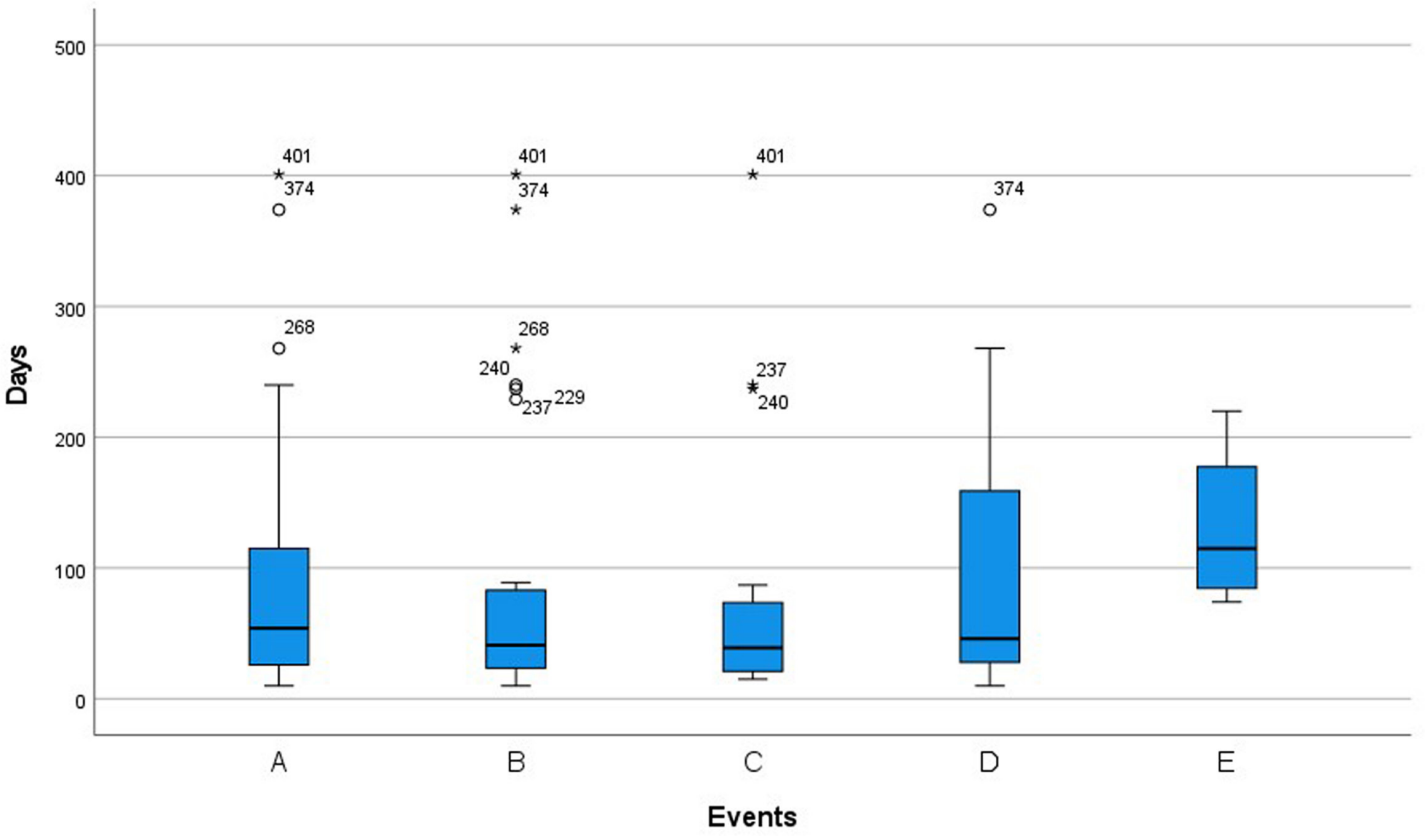
Time to immune checkpoint inhibitor‐related cardiac adverse events from the first immune checkpoint inhibitor‐based therapy. (A) All cardiac adverse events; (B) all myocarditis; (C) severe myocarditis; (D) non‐severe myocarditis; (E) non‐myocarditis. ○: outlier; *: extremum.

We included 44 patients from 11 different centers including 35 myocarditis, 5 heart failure, 3 arrhythmias, and 1 myocardial infarction. The median age of included patients was 64 (IQR: 57–69) years old. Patients were predominantly male (56.8%, *n* = 25 of 44). Fifteen patients (34.1%) received ICIs for the treatment of lung cancer, fourteen (31.8%) for gastrointestinal cancer, seven (15.9%) for urologic neoplasms, four (9.1%) for gynecologic cancer, and 4 (9.1%) for other cancers. The most common types of ICIs are pembrolizumab (*n* = 11, 25%) and camrelizumab (*n* = 11, 25%), followed by sintilimab (*n* = 7, 15.9%), tislelizumab (*n* = 4, 9.1%), and nivolumab (*n* = 3, 6.8%). The median time to ICI‐associated cardiovascular adverse events was 71 (IQR: 29–97) days (Figure 1). Eight patients (18.2%) were treated with anti‐PD1 monotherapy, twenty‐one (47.7%) combined with chemotherapy, twelve (27.3%) combined with targeted therapy, and three (6.8%) combined with chemotherapy and targeted therapy. The ECG was abnormal in 29 (69.5%) patients, and the transthoracic echocardiography (TTE) showed that the LVEF decreased to lower than 50% in 9 out of 44 (20.5%) patients. Patients mainly first presented with flu‐like symptoms (*n* = 13, 29.5%) and palpitations (*n* = 13, 29.5%). Twenty‐seven of 44 patients had at least one comorbidity, and hypertension was the most common (*n* = 17, 38.6%) (Table 1).

**TABLE 1:** Description of patients with ICIs‐induced cardiac adverse events.

Characteristics	Total
Number of patients	44
Age, median (IQR)	64 (57–69)
Male (%)	25 (56.8)
Cancer type (%)	
Lung cancer	15 (34.1)
Gastrointestinal cancer	14 (31.8)
Urologic neoplasms	7 (15.9)
Gynecologic cancer	4 (9.1)
Others	4 (9.1)
ICIs type (%)	
Pembrolizumab	11 (25)
Camrelizumab	11 (25)
Sintilimab	7 (15.9)
Tislelizumab	4 (9.1)
Nivolumab	3 (6.8)
Others	8 (18.2)
Time to cardiac adverse events, median (IQR)	71 (29–97)
Abnormal ECG	29 (65.9)
Decreased LVEF	9 (20.5)
First symptoms (%)	
Flu‐like symptoms	13 (29.5)
Palpitations	13 (29.5)
Chest tightness	10 (22.7)
Dyspnea	9 (20.5)
Chest pain	5 (11.4)
Others	10 (22.7)
Combined treatment (%)	
Chemotherapy	21 (47.7)
Targeted therapy	12 (27.3)
Chemotherapy+ targeted therapy	3 (6.8)
Past comorbidities (%)	
Hypertension	17 (38.6)
Coronary heart disease	10 (22.7)
Diabetes mellitus	8 (18.2)
Arrhythmia	2 (4.5)
Cardiomyopathy	1 (2.3)
Autoimmune disease	1 (2.3)

Abbreviations: ECG, electrocardiogram; ICIs, immune checkpoint inhibitors; IQR, interquartile range; LVEF, left ventricular ejection fraction.

### 3.2 Comparison between myocarditis patients versus non‐myocarditis patients

Most myocarditis patients (28 out of 35) and non‐myocarditis patients (8 of 9) received combination anti‐PD1‐based therapy. The most common non‐cardiac immune‐related adverse event between these two groups is transaminitis (Table 2). When we compared the myocarditis patients (*n* = 35) versus non‐myocarditis patients (*n* = 9) (Table 2), they presented higher cTnI levels (median of 25.1‐fold the upper limit vs. 0.27‐fold, *p* < 0.001), higher CK levels (median of 6‐fold the upper limit vs. 0.3‐fold, *p* = 0.003), higher CK‐MB levels (median of 4.9‐fold the upper limit vs. 0.7‐fold, *p* = 0.013), and shorter time to the incidence of adverse cardiovascular events (median of 41 days vs. median of 115 days, *p* = 0.022) after ICI treatment (Figure 1). There was no statistically significant difference in age, percentage of males, baseline Eastern Cooperative Oncology Group (ECOG) score, cancer treatment, NT‐proBNP, D‐dimer, NLR, conduction disorder on ECG (including bundle branch blocks and atrioventricular blocks), decreased LVEF on TTE, Albumin/globulin, AST/ALT, LDL/HDL, non‐cardiac immune‐related adverse events, or past comorbidities between the two groups (Table 2).

**TABLE 2:** Comparison between myocarditis patients versus non‐myocarditis patients.

Characteristics	Myocarditis patients (*n* = 35)	Non‐myocarditis patients (*n* = 9)	*p* value
Age, median (IQR)	64 (56.8–71.3)	66 (59–68.5)	0.988
Male	19 (54.3)	6 (66.7)	0.771
Baseline ECOG score, median (IQR)	1 (1–3)	2 (0.5–3)	0.892
Anti‐PD1 monotherapy	7 (20)	1 (11.1)	0.895
Combination anti‐PD1‐based therapy			
Chemotherapy	17 (48.6)	4 (44.4)	1.000
Targeted therapy	8 (22.9)	4 (44.4)	0.380
Chemotherapy+ targeted therapy	3 (8.6)	0 (0)	1.000
Cardiac biomarkers (median, upper limit, IQR)			
cTnI	25.1 (6.1–192)	0.27 (0.17–0.33)	**<0.001**
NT‐proBNP	3.9 (1.1–15.3)	7.5 (0.9–37.3)	0.915
CK	6 (0.5–28.1)	0.3 (0.2–3.6)	**0.003**
CK‐MB	4.9 (1.4–7.8)	0.7 (1.6–2.8)	**0.013**
D‐dimer (median, upper limit, IQR)	2.2 (0.9–5.7)	2.2 (1.5–8.5)	0.518
NLR, median (IQR)	8.8 (5.1–14.2)	3.7 (3.3–15.9)	0.117
Conduction disorders on ECG	22 (62.9)	6 (66.7)	1.000
TTE LVEF <50%	8 (26.7)	1 (11.1)	0.603
Albumin/globulin, median (IQR)	1.4 (1.1–1.7)	1.4 (1.2–1.7)	0.565
AST/ALT, median (IQR)	1.2 (0.9–2.1)	1 (0.5–1.6)	0.210
LDL/HDL, median (IQR)	2.9 (2.3–3.3)	2 (1.1–4.8)	0.351
Time to cardiac irAEs, median (IQR)	41 (22–87)	115 (80–191)	**0.022**
Non‐cardiac irAEs			
Transaminitis	24 (68.6)	5 (55.6)	0.734
Myositis	17 (48.6)	2 (22.2)	0.296
Thyroiditis	10 (28.6)	4 (44.4)	0.610
Myasthenia gravis	11 (31.4)	2 (22.2)	0.896
Nephritis	7 (20)	1 (11.1)	0.895
Pneumonitis	9 (25.7)	0 (0)	0.214
Dermatitis	3 (8.6)	2 (22.2)	0.267
Gastroenteritis	5 (14.3)	0 (0)	0.566
Past comorbidities			
Hypertension	12 (34.3)	5 (55.6)	0.432
Coronary heart disease	7 (20)	3 (33.3)	0.685
Diabetes mellitus	6 (17.1)	2 (22.2)	1.000

Abbreviations: ALT, alanine transaminase; AST, aspartate transaminase; CK, creatine kinase; CK‐MB, creatine kinase isoenzyme‐MB; cTnI, cardiac troponin I; ECG, electrocardiogram; ECOG, Eastern Cooperative Oncology Group; HDL, high‐density lipoprotein; IQR, interquartile range; irAEs, immune‐related adverse events; LDL, low‐density lipoprotein; LVEF, left ventricular ejection fraction; NLR, neutrophil to lymphocyte ratio; NT‐proBNP, N‐terminal pro‐B‐type natriuretic peptide; PD‐1, programmed cell death protein‐1; TTE, transthoracic echocardiography. Bold values mean statistical differences are significant.

### 3.3 Univariate and multivariate logistic regression of the risk factors affecting the incidence of ICI‐associated myocarditis

To explore the risk factors for the development of ICI‐associated myocarditis, we performed logistic regression on indicators that differed between the myocarditis and non‐myocarditis groups. Univariate logistic regression showed that higher CK‐MB after ICI treatment (unadjusted odds ratio [OR]: 1.513, 95% confidence interval [CI]: 1.149–1.993, *p* = 0.005) and time to immune‐related adverse events (unadjusted OR: 1.006, 95% CI: 1.000–1.012, *p* = 0.039) were associated with the higher risk of ICI‐associated myocarditis. After adjustment for age, sex, hypertension, coronary heart disease, diabetes mellitus, and low‐density lipoprotein/high‐density lipoprotein, the result of CK‐MB remained significant (adjusted OR: 1.775, 95% CI: 1.055–2.984, *p* = 0.031). However, for time to immune‐related adverse events, no significant association was found (adjusted OR: 0.995, 95% CI: 0.986–1.003, *p* = 0.244) (Table 3).

**TABLE 3:** Logistic regression analysis of risk factors affecting the incidence of myocarditis.

Factors	Univariate analysis			Multivariate analysis		
	OR	95% CI	*p*	OR	95% CI	*p*
CK	1.649	0.997–2.725	0.051	1.310	0.801–2.144	0.282
CK‐MB	1.513	1.149–1.993	0.005	1.775	1.055–2.984	0.031
Time to cardiac irAEs	1.006	1.000–1.012	0.039	0.995	0.986–1.003	0.244
cTnI	2.334	0.823–6.621	0.111	7.923E+21	0.000–9.740E+239	0.844

*Note*: Adjusting risk factors: age, sex, hypertension, coronary heart disease, diabetes mellitus, and low‐density lipoprotein/high‐density lipoprotein.

Abbreviations: CI, confidence interval; CK, creatine kinase; CK‐MB, creatine kinase isoenzyme‐MB; cTnI, cardiac troponin I; irAEs, immune‐related adverse events; OR, odds ratio.

### 3.4 Comparison between severe myocarditis patients versus non‐severe myocarditis patients

Most severe myocarditis patients (17 out of 21) and non‐severe myocarditis patients (12 out of 14) received combination anti‐PD1‐based therapy. The most common non‐cardiac immune‐related adverse event between these two groups is transaminitis (Table 4). When we compared the severe myocarditis patients (*n* = 21) versus non‐severe myocarditis patients (*n* = 14) (Table 4), they presented higher cTnI levels (median of 63.9‐fold the upper limit vs. 8.8‐fold, *p* = 0.013), higher NT‐proBNP levels (median of 5.1‐fold the upper limit vs. 1.2‐fold, *p* = 0.031), higher CK levels (median of 9.1‐fold the upper limit vs. 0.4‐fold, *p* = 0.018), higher CK‐MB levels (median of 6.8‐fold the upper limit vs. 1.3‐fold, *p* = 0.026), and higher NLR levels (median of 11.4‐fold the upper limit vs. 6.8‐fold, *p* = 0.016) after ICI treatment. There was no statistically significant difference in age, percentage of males, ECOG score, cancer treatment, D‐dimer, conduction disorder on ECG, decreased LVEF on TTE, Albumin/globulin, AST/ALT, LDL/HDL, time to the incidence of myocarditis, non‐cardiac immune‐related adverse events, past comorbidities, myocarditis treatment and mortality between the two groups (Table 4). However, although there was no statistical difference, severe myocarditis patients had a shorter median time to onset of myocarditis (39 days vs. 46 days) (Figure 1) and showed higher mortality (42.9% vs. 28.6%).

**TABLE 4:** Comparison between severe myocarditis patients versus non‐severe myocarditis patients.

Characteristics	Severe myocarditis patients (*n* = 21)	Non‐severe myocarditis patients (*n* = 14)	*p* value
Age, median (IQR)	67 (59–73)	61.5 (56–68)	0.151
Male	12 (57.1)	8 (57.1)	1.000
Baseline ECOG score, median (IQR)	2 (1–3.5)	1 (1–2)	0.164
Anti‐PD1 monotherapy	4 (19)	2 (14.3)	1.000
Combination anti‐PD1‐based therapy			
Chemotherapy	8 (38.1)	9 (64.3)	0.129
Targeted therapy	6 (28.6)	2 (14.3)	0.431
Chemotherapy+ targeted therapy	3 (14.3)	1 (7.1)	0.635
Cardiac biomarkers (median, upper limit, IQR)			
cTnI	63.9 (13.7–225.8)	8.8 (2.2–34.7)	**0.013**
NT‐proBNP	5.1 (2.3–21.5)	1.2 (0.6–5.4)	**0.031**
CK	9.1 (2.2–36.7)	0.4 (0.3–13.4)	**0.018**
CK‐MB	6.8 (3–11.4)	1.3 (0.6–6.7)	**0.026**
D‐dimer (median, upper limit, IQR)	2.7 (0.9–5.6)	1.8 (0.6–10.1)	0.851
NLR, median (IQR)	11.4 (7–18.9)	6.8 (4.5–8.9)	**0.016**
TTE LVEF <50%	7 (33.3)	1 (7.1)	0.307
Albumin/globulin, median (IQR)	1.4 (0.9–1.8)	1.3 (1.3–1.6)	0.882
AST/ALT, median (IQR)	1.4 (1–2.2)	1.2 (0.8–1.6)	0.273
LDL/HDL, median (IQR)	3 (2.4–3.6)	2.8 (2.2–3.2)	0.364
Time to myocarditis, median (IQR)	39 (20–79)	46 (27–194)	0.516
Non‐cardiac irAEs			
Transaminitis	17 (81)	7 (50)	0.119
Myositis	12 (57.1)	5 (35.7)	0.214
Thyroiditis	5 (23.8)	5 (35.7)	0.703
Myasthenia gravis	8 (38.1)	3 (21.4)	0.504
Nephritis	4 (19)	3 (21.4)	1.000
Pneumonitis	7 (33.3)	2 (14.3)	0.385
Dermatitis	1 (4.8)	2 (14.3)	0.551
Gastroenteritis	4 (19)	1 (7.1)	0.627
Past comorbidities			
Hypertension	7 (33.3)	5 (35.7)	1.000
Coronary heart disease	4 (19)	3 (21.4)	1.000
Diabetes mellitus	5 (23.8)	2 (14.3)	0.676
Myocarditis treatment			
Glucocorticoid monotherapy	9 (42.9)	5 (35.7)	0.673
Glucocorticoid+IG	6 (28.6)	0 (0)	0.061
Glucocorticoid+MMF+IG	1 (4.8)	3 (21.4)	0.279
Death	9 (42.9)	4 (28.6)	0.392

Abbreviations: ALT, alanine transaminase; AST, aspartate transaminase; CK, creatine kinase; CK‐MB, creatine kinase isoenzyme‐MB; cTnI, cardiac troponin I; ECG, electrocardiogram; ECOG, Eastern Cooperative Oncology Group; HDL, high‐density lipoprotein; IG, immunoglobulin; IQR, interquartile range; irAEs, immune‐related adverse events; LDL, low‐density lipoprotein; LVEF, left ventricular ejection fraction; MMF, mycophenolate mofetil; NLR, neutrophil to lymphocyte ratio; NT‐proBNP, N‐terminal pro‐B‐type natriuretic peptide; PD‐1, programmed cell death protein‐1; TTE, transthoracic echocardiography. Bold values mean statistical differences are significant.

### 3.5 Univariate and multivariate logistic regression of the risk factors affecting the incidence of ICI‐associated severe myocarditis

To explore the risk factors for the development of ICI‐associated severe myocarditis, we performed logistic regression. Univariate logistic regression showed that higher cTnI (unadjusted OR: 1.013, 95% CI: 1.000–1.025, *p* = 0.047) and higher CK‐MB (unadjusted OR: 1.121, 95% CI: 1.002–1.254, *p* = 0.046) after ICI treatment were associated with the higher risk of ICI‐associated severe myocarditis. After adjustment for age, sex, hypertension, coronary heart disease, diabetes mellitus, and low‐density lipoprotein/high‐density lipoprotein, we found that higher cTnI (adjusted OR: 1.019, 95% CI: 1.002–1.037, *p* = 0.029) and higher NLR (adjusted OR: 1.89, 95% CI: 1.026–3.483, *p* = 0.041) after ICI treatment were the independent risk factors for the development of ICI‐associated severe myocarditis, but there was no significant association between NT‐proBNP (adjusted OR: 1.006, 95% CI: 0.993–1.018, *p* = 0.368), CK (adjusted OR: 1.029, 95% CI: 0.988–1.071, *p* = 0.172), CK‐MB (adjusted OR: 1.188, 95% CI: 0.967–1.461, *p* = 0.101), or time to the onset of myocarditis (adjusted OR: 0.997, 95% CI: 0.989–1.005, *p* = 0.424) and ICI‐associated severe myocarditis (Table 5).

**TABLE 5:** Logistic regression analysis of risk factors affecting the incidence of severe myocarditis.

Factors	Univariate analysis			Multivariate analysis		
	OR	95% CI	*p*	OR	95% CI	*p*
cTnI	1.013	1.000–1.025	0.047	1.021	1.002–1.039	0.03
NLR	1.072	0.993–1.157	0.076	1.890	1.026–3.483	0.041
NT‐proBNP	1.006	0.995–1.017	0.291	1.006	0.993–1.018	0.368
CK	1.030	0.996–1.064	0.083	1.029	0.988–1.071	0.172
CK‐MB	1.121	1.002–1.254	0.046	1.188	0.967–1.461	0.101
Time to myocarditis	1.001	0.996–1.006	0.712	0.997	0.989–1.005	0.424

*Note*: Adjusting risk factors: age, sex, hypertension, coronary heart disease, diabetes mellitus, and low‐density lipoprotein/high‐density lipoprotein.

Abbreviations: CI, confidence interval; CK, creatine kinase; CK‐MB, creatine kinase isoenzyme‐MB; cTnI, cardiac troponin I; NLR, neutrophil to lymphocyte ratio; NT‐proBNP, N‐terminal pro‐B‐type natriuretic peptide; OR, odds ratio.

### 3.6 The diagnostic ability of biomarkers to distinguish ICI‐associated myocarditis and severe myocarditis

**FIGURE 2:**
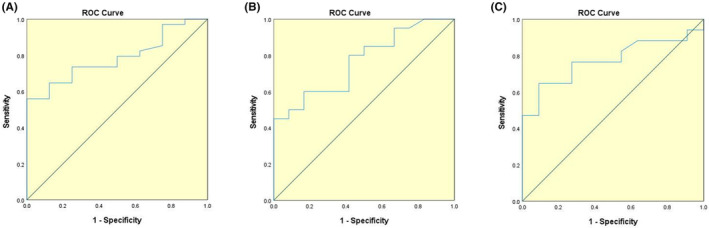
Receiver operating characteristic curve for: (A) creatine kinase isoenzyme‐MB (CK‐MB) between myocarditis patients and non‐myocarditis patients; (B) cardiac troponin I between severe myocarditis patients and non‐severe myocarditis patients; (C) neutrophil to lymphocyte ratio (NLR) between severe myocarditis patients and non‐severe myocarditis patients.

To explore any predictive ability for the incidence of ICI‐associated myocarditis, we conducted ROC curve analyses for the biomarkers associated with ICI‐associated myocarditis, showing an area under curve (AUC) of 0.785 (95% CI: 0.642 to 0.928, *p* = 0.013) (Figure 2A) for CK‐MB. Hosmer–Lemeshow test value was *p* = 0.898 (>0.05). When selecting a cut‐off value of 5.28‐fold increase beyond the upper limit established in the laboratory, we found a specificity of 100% and a sensitivity of 55.9%. Concerning severe myocarditis and non‐severe myocarditis groups, the ROC analysis demonstrated an AUC of 0.765 for cTnI (95% CI: 0.601 to 0.929, *p* = 0.013) (Figure 2B) and 0.773 for NLR (95% CI: 0.597 to 0.948, *p* = 0.016) (Figure 2C). Hosmer–Lemeshow test values were *p* = 0.830 and *p* = 0.266 (both >0.05). For cTnI, choosing a threshold corresponding to a 143.575‐fold the laboratory upper limit yielded a specificity of 100% and a sensitivity of 45%. While for NLR, when choosing the value of 9.935, we found a sensitivity of 64.7% and a specificity of 90.9%.

## 4 DISCUSSION

Herein, we investigated a retrospective cohort of 44 cases of cardiovascular adverse events in patients treated by ICIs. The main findings are: (i) compared with ICI‐induced non‐myocarditis cardiovascular adverse events, ICI‐associated myocarditis patients presented with higher cTnI levels, higher CK levels, higher CK‐MB levels, and shorter time to the incidence of adverse cardiovascular events after ICI treatment; (ii) CK‐MB is the independent risk factor of the incidence of ICI‐associated myocarditis; (iii) compared with ICI‐associated non‐severe myocarditis, severe myocarditis patients presented with higher cTnI levels, higher NT‐proBNP levels, higher CK levels, higher CK‐MB levels, and higher NLR levels after ICI treatment; (iv) cTnI and NLR were the independent risk factors of the development of ICI‐associated severe myocarditis; (v) the ROC curve analyses demonstrated that CK‐MB exhibited favorable predictive value in the diagnosis of ICI‐associated myocarditis, and cTnI and NLR demonstrated a promising predictive utility for the identification of severe myocarditis.

The cardiovascular toxicities associated with ICIs encompass a spectrum of pathological conditions. This study describes a widening spectrum of ICI‐related cardiovascular complications, including myocarditis, arrhythmias, congestive heart failure, acute coronary syndrome, and so forth. ICI‐induced myocarditis, in particular, often presents with non‐specific initial symptoms such as fatigue, palpitations, and dyspnea. As the condition progresses, patients may exhibit orthopnea, lower extremity edema, and, in severe cases, clinical manifestations may culminate in sudden cardiac death.^10^, ^15^ The presence of arrhythmias was identified as a significant risk factor that influence the prognosis of ICI‐associated cardiac adverse events.^16^

In our study, the median time to cardiovascular adverse events was 71 (IQR: 29–97) days after the initiation of ICIs, suggesting that these events are probably related to ICI treatment and are part of the ICI‐associated cardiovascular adverse events. We posit that the findings reported are of high relevance to the realm of cardio‐oncology. In our study, most of the myocarditis cases occurred within 1–3 months after ICI initiation. The onset of myocarditis was shorter than the median time to the onset of all cardiovascular events (41 days vs. 71 days) and non‐myocarditis adverse events (41 days vs. 115 days). Coustal et al.^17^ found that the median time to the onset of myocarditis was 39 days. A retrospective pooled analysis including 18 myocarditis patients showed that patients exhibited myocarditis following a median of two administrations of ICI (with an IQR of 1–3.5 doses), and this occurred at a median of 35 days (with an IQR of 19–80 days) after the initial administration of ICIs.^14^ A recent pooled analysis found the early onset of myocarditis within the first two cycles of ICIs therapy was significantly associated with worse outcomes.^16^ In our study, although the time of onset of myocarditis was not a risk factor for severe myocarditis, we found that severe myocarditis occurred earlier than non‐severe myocarditis (39 days vs. 46 days). These results suggest that myocarditis is characterized by an abrupt onset, especially severe myocarditis, and is associated with rapid progression and high mortality. Early clinical intervention is required to improve the prognosis of patients.

Endomyocardial biopsy is widely regarded as the gold‐standard technique for the diagnosis of myocarditis. Cardiac magnetic resonance imaging stands as the primary non‐invasive diagnostic modality for myocarditis. While contemporary algorithms suggest the acquisition of endomyocardial biopsy and cardiac magnetic resonance imaging when encountering suspected cases of ICI‐associated myocarditis, it is worth noting that these procedures may not be universally available across all medical centers. The use of endomyocardial biopsy is limited by the fact that it is an invasive test. Cardiac magnetic resonance incurs substantial costs, necessitates the administration of contrast agents via injection, entails a prolonged examination duration, and is encumbered by contraindications, rendering it challenging to employ as a customary clinical diagnostic procedure. In certain instances, they could fail to uncover a potential diagnosis, even in situations where clinical suspicion is considerably high. Hence, there is a great need for simpler modalities, such as elevated cardiac markers, for the diagnosis of myocarditis.

We found some prognostic factors, such as higher cTnI levels, higher CK levels, higher CK‐MB levels after ICI treatment, and shorter time to the incidence of adverse cardiovascular events that were correlated with ICI‐associated myocarditis. Higher sensitivity for ICI myocarditis of CK‐MB compared with CK or cTnI was observed in our study. Puzanov et al. found that higher cTnI (*p* = 0.037) and CK‐MB (*p* = 0.034) were associated with progression to severe ICI‐associated myocarditis. Higher cTnI (*p* = 0.016), CK‐MB (*p* = 0.034), and CK (*p* = 0.013) levels were correlated with increased mortality.^18^ Interestingly, our study showed that cTnI could be identified as a diagnostic tool for ICI‐induced severe myocarditis, which has a favorable predictive value than NT‐proBNP, CK, CK‐MB. This is consistent with the results of a previous case series study.^17^ It has also been suggested that patients with higher troponin have a higher risk of cardiac adverse events.^16^ Until now, the majority of documented cases of ICI‐induced myocarditis in the existing literature have been detected through the use of cTnI assays. These assays are prevalent and accessible from numerous suppliers and are frequently favored over other cardiac biomarkers due to recent ICI myocarditis diagnostic guidelines.^10^ Further studies are needed to confirm the diagnostic value of CK‐MB in ICI‐associated myocarditis.

Our study found that NLR elevated in patients with severe myocarditis and demonstrated that NLR was an independent predictor of ICI‐associated severe myocarditis. This is consistent with previous findings. Mirna et al.^19^ showed a correlation with NLR and the length of hospital stay in myocarditis patients. Previous studies have shown that NLR increased in patients with ICI‐induced myocarditis and that elevated NLR is associated with major adverse cardiovascular events related to ICI therapy.^20^, ^21^, ^22^ Our results further confirm the association between NLR and cardiovascular events as reported in previous studies. NLR reflects inflammatory activity, which may be as a possible mediator for severe myocarditis in cancer patients receiving ICIs.

No significant correlation was observed between combination anti‐PD1‐based therapy and the severity of cardiovascular toxicities in our study, despite existing literature suggesting an increased incidence rate associated with ICI combination therapy. A recent study arising from the JAVELIN Renal 101 clinical trial has identified a higher prevalence of LVEF reduction in patients who received a combination therapy of avelumab and axitinib compared to those receiving sunitinib as a monotherapy (8.5% vs. 1.6%), indicating a more pronounced additive influence in the cohort treated with the combination regimen.^23^ A recent meta‐analysis found that co‐administered ICI with anti‐VEGF agents can increase the risk of a grade 3–4 hypertension, a grade 3–4 cardiac disorder, an all‐grade cardiac disorder, a grade 3–4 arterial thromboembolic event, an all‐grade arterial thromboembolic event, and a grade 3–4 venous thrombotic event compared to anti‐VEGF agents alone. In addition, patients receiving ICI combined with anti‐VEGF agents are more likely to experience fatal cardiovascular events compared to those receiving anti‐VEGF alone.^24^ This discrepancy between the results of the above studies and ours might be attributed to the enhanced monitoring of patients undergoing combination therapy in our study. In addition, combination therapy with different types of ICIs may increase the mortality rate. Salem et al. found that within the cohort of myocarditis cases, the incidence of mortality was notably higher in individuals undergoing ICI combination therapy compared to those receiving ICI monotherapy (65.6% vs. 44.4%, *p* = 0.04).^25^ However, since most patients included in our study received a single ICI treatment, it was not available to compare the effects of ICI combination therapy and monotherapy on the occurrence of adverse cardiovascular events.

In earlier observations drawn from pharmacovigilance databases and multicenter registries, a notable mortality rate within the range of 25%–50% had been reported for cases of myocarditis.^26^ In our study, the mortality associated with myocarditis induced by ICI was found to be comparatively lower than the figures reported in prior pharmacovigilance analyses and other real‐world data compilations. This discrepancy can likely be attributed to a confluence of multiple contributing factors. The cardio‐oncology department at the collaborative medical centers for this study performed a rapid assessment of patients, leading to early diagnosis and treatment. The majority of patients diagnosed with myocarditis, or those exhibiting a high level of clinical suspicion and clinical instability, initiated a regimen involving high‐dose steroids alongside the interruption of ICI treatment. The existing protocol employed within our healthcare service followed this course: intravenous administration of methylprednisolone at a dosage ranging from 500 to 1000 mg, administered once daily for a minimum of 3 days, with continuation until the troponin level stabilizes and any concurrent clinical complications, such as heart failure or ventricular arrhythmias, have resolved. The prompt administration of high‐dose steroids for ICI‐myocarditis is purported to mitigate the occurrence of major adverse cardiovascular events and cardiovascular mortality,^27^ potentially contributing to the observed low mortality rates within this management pathway.

Up to date, there is an absence of established, standardized protocols for the medical management of ICI‐cardiovascular adverse events. For cancer patients experiencing ICI‐related cardiovascular complications, it is customary to temporarily discontinue ICI therapy until the confirmation of a diagnosis. This is of significant importance, primarily due to the fact that myocarditis stands as a relative contraindication for the continuation of ICI therapy.^28^ ICI‐associated myocarditis is managed through discontinuation of ICI therapy and the commencement of high‐dose intravenous corticosteroids. Supplementary treatment options encompass anti‐thymocyte globulin (anti‐CD3 antibody), alemtuzumab (anti‐CD52 antibody), and abatacept (a CTLA‐4 agonist). However, it is noteworthy that there is a lack of clinical trial evidence supporting the efficacy of these therapeutic interventions.^29^, ^30^ In contrast, in cases where patients encounter other ICI‐related cardiac issues, such as heart failure, atrial fibrillation, and acute myocardial infarction, the prevailing practice allows for the resumption of ICI treatment subsequent to appropriate intervention and management. This approach aligns with the existing paradigm of adhering to standard cardiology guidelines when addressing these other ICI‐associated cardiac toxicities.^31^ Furthermore, in the context of encountering intricate immune‐related adverse events scenarios, such as ICI‐cardiovascular adverse events concurrent with multisystem non‐cardiac immune‐related adverse events in real‐world clinical practice, it is imperative to underscore the importance of forming interdisciplinary teams. Compared with patients who developed only myocarditis, concurrent of myositis was a risk factor of severe myocarditis.^16^ The observation that almost 70% of patients in our cohort of ICI myocarditis present with transaminitis and half of patients present with ICI myositis underscores the prevailing association of ICI myocarditis with the broader spectrum of systemic ICI myotoxicity. The interdisciplinary teams should comprise cardiologists, oncologists, internists, and professionals from various relevant disciplines, with the aim of facilitating the judicious and prompt management of patient requirements.^14^

### 4.1 Clinical significance

We anticipate that CK‐MB, cTnI, and NLR hold promise for forecasting the emergence of clinically substantial ICI‐induced myocarditis and severe myocarditis. This approach has the potential to facilitate the development of novel monitoring protocols, thereby streamlining existing non‐evidence‐based surveillance practices. This study also serves to highlight the existence of additional cardiovascular complications associated with ICIs, extending beyond the scope of myocarditis. Notably, heart failure and tachyarrhythmias, such as atrial fibrillation, were identified as some of the more prevalent complications. Moreover, of substantial clinical significance is the relatively less frequent yet critical manifestation of ICI‐related ischaemic heart disease, encompassing scenarios such as acute myocardial infarction necessitating urgent percutaneous coronary intervention. Our study is expected to further enrich the knowledge of clinicians about ICI‐related cardiovascular toxicities.

### 4.2 Study limitations

The present study has several limitations. First, the limited sample size employed in this study curtails the statistical power of our findings. Particularly in the examination of risk factors contributing to ICI‐reduced adverse cardiac events, there is a potential for certain risk factors to remain undetected. Second, some of the patients included in this study were contingent upon referrals from oncology teams, potentially introducing a referral bias. In cases where cardiovascular toxicities have not been recognized, patients might not be referred, consequently constraining the scope of conclusions that can be drawn about cardiovascular adverse events within the broader population of individuals undergoing ICI treatment. In addition, the number of endomyocardial biopsies conducted was limited, making statistical comparisons between histological and clinical findings unfeasible. Finally, certain specific data points, such as the initiation date of ICI treatment and the number of treatment cycles completed prior to the onset of adverse events, were not available for all cases under consideration.

## 5 CONCLUSIONS

Elevated CK‐MB after ICI treatment independently contributes to the risk of developing ICI‐associated myocarditis, whereas higher cTnI and NLR after ICI treatment serve as independent risk factors for the progression to severe ICI‐associated myocarditis. CK‐MB, cTnI and NLR have shown potential as reliable predictors for diagnosing ICI‐associated myocarditis and severe myocarditis.

## AUTHOR CONTRIBUTIONS

**Yi Zheng:** Conceptualization (lead); data curation (lead); formal analysis (lead); methodology (lead); software (lead); validation (lead); visualization (lead); writing – original draft (lead). **Ziliang Chen:** Investigation (equal); resources (equal). **Wenhua Song:** Investigation (equal). **Yu Xu:** Investigation (equal). **Zhiqiang Zhao:** Resources (equal). **Yihong Sun:** Resources (equal). **Yuanyuan Wang:** Resources (equal). **Xuhong Geng:** Resources (equal). **Jun Zhao:** Resources (equal). **Xiaowei Zhang:** Resources (equal). **Yanmin Xu:** Resources (equal). **Jeffrey Shi Kai Chan:** Writing – review and editing (equal). **Gary Tse:** Writing – review and editing (equal). **Guangping Li:** Supervision (equal). **Lili Hong:** Project administration (equal); supervision (equal). **Tong Liu:** Funding acquisition (lead); project administration (equal); supervision (equal).

## FUNDING INFORMATION

This work was funded by the National Natural Science Foundation of China (82170327, 82370332 to TL) and Tianjin Key Medical Discipline (Specialty) Construction Project (TJYXZDXK‐029A, TJWJ2022XK013).

## CONFLICT OF INTEREST STATEMENT

The authors declare that they have no conflict of interests.

## ETHICS STATEMENT

This study was approved by the Ethics Committee of the Second Hospital of Tianjin Medical University (KY2018K049).

## PATIENT CONSENT STATEMENT

Informed consent was obtained for each participant.

## Data Availability

All relevant data are within the manuscript.
